# Trends and trajectories in employee green behavior research

**DOI:** 10.3389/fsoc.2024.1486377

**Published:** 2024-11-20

**Authors:** Azlan Ali, Huang Juan

**Affiliations:** ^1^Graduate School of Management, Management and Science University, Shah Alam, Malaysia; ^2^Guangxi Vocational Normal University, Nanning, China

**Keywords:** employee green behavior, green innovation, sustainability, bibliometric analysis, research trends, research trajectories

## Abstract

With environmental protection awareness increasing, green innovation has become a key way for enterprises to achieve sustainable development. Research trends on employee green behavior are an important basis for formulating green behavior incentive measures and a key foundation for further exploring green innovation. However, due to the large amount of literature on employee green behavior, obtaining research trends directly related to employee green behavior takes time and effort. To solve this problem, this paper takes the relevant published literature on research on employee green behavior from 2009 to 2024 as the research object. It uses CiteSpace software to study the research trends of employee green behavior from the number change analysis of published literature, distribution region analysis of published literature, influence analysis of main authors of published literature, keyword analysis, and high-frequency word analysis. The research results show that the publication of literature on employee green behavior has been steadily increasing since 2018, and the relevant research mainly focuses on the impact of green behavior motivations, green behavior emotional factors, green behavior performance results, green self-energy efficiency, and other aspects on employee green behavior. Based on the research results, further summaries and suggestions are given to provide references for the subsequent related research in this paper.

## 1 Introduction

The deterioration of the environment and the scarcity of resources have promoted global attention to environmental management or green management, and green has become the norm. Green human resource management is a management concept and management model in the new era. Its main task is to achieve the three major harmonies of mental harmony, human harmony and ecological harmony among employees within the enterprise by adopting management methods that are in line with the “green” concept, thereby bringing comprehensive benefits that unify economy, society and ecology to the enterprise. Carrying out green management has become a key way for companies to pursue sustainability. As policy executors in the enterprise’s green management process, employee behavior determines the effectiveness of enterprise’s green management. Green management requires employees to not only have a strong awareness of environmental protection, but also actively use green behaviors to promote high-quality development of enterprises. Employee green behavior has become a key factor in promoting sustainable development of enterprises.

As an important carrier of primary productivity, employees have more fundamental significance for green development than the material resources of the enterprise. In recent years, research on employee green behavior has become the core content of green human resource management. Research on employee green behavior also advocates people-oriented, unifying employee individual goals and corporate goals as much as possible, and then forming a collaborative and harmonious development relationship among employees, enterprises and society (Chien, 2024). As major participants in corporate activities, employee green behavior has an important impact on the development of enterprise. Employees play a central role in the green development of enterprises, and their green behavior is an important way for enterprises to achieve sustainable development (Nguyen et al., 2023; Khan et al., 2023). It can be seen that employee green behavior has become a crucial factor for the enterprise to achieve the sustainable development (Malokani et al., 2023).

Research on employee green behavior has become a key interdisciplinary research focus. Key issues worthy of further study include how to use employee green behavior to promote corporate green management, what factors interact with employee green behavior to form effective mechanisms, and what incentive results such behaviors can produce. Research on employee green behavior spans multiple disciplines, and the number of annual publications on the topic is changing rapidly (Shahzad et al., 2023; Han et al., 2023; Zhang et al., 2024; Wells et al., 2024). Research on employee green behavior is important in promoting enterprises to achieve social and ecological benefits (Amini et al., 2024). The research and practice of employee green behavior, especially under the guidance of green development concepts, is of far-reaching significance to enterprises and society (Chand et al., 2023). Because employees are one of the most important resources of enterprises, their behaviors directly affect the environmental protection impact of enterprises. To reduce environmental pollution in the production process and obtain much more economic benefits, it is very necessary to study measures that improve employee behavior (Różańska-Bińczyk et al., 2024; Wang et al., 2024).

Research on employee green behavior has attracted the attention of management, economics, psychology, and sociology. Research hotspots and trends of employee green behavior have become a key basis for enterprises to formulate the green behavior incentive measures. How to use literature statistical analysis tools to reveal research trends of employee green behavior has become an important topic. For scholars and corporate management, obtaining the research trends of employee green behavior is very significant. It will effectively help establish effective measures for green human resources management.

The main purposes of this study are to analyze trajectories, discover hotspots, reveal trends and provide insights for future research in employee green behavior research. This study uses bibliometric analysis to investigate publishing trends, regional distribution, influential authors and keyword co-occurrences to map the pattern of employee green behavior research. The study is carried out from the following aspects:

1. To understand trajectories and trends of employee green behavior research, the publications from 2009 to 2024 was taken as object to create chart analysis.
2. To analyze the scholars’ attention on employee green behavior research, the publications of major journals are used to carry out comparative analysis.
3. To discover the concern and importance of green employee behavior in different countries, the distribution of publication region of published literature is studied.
4. To find high-impact authors in the research field of employee green behavior, the literature number and the literature half-life of authors are taken to create analysis.
5. To discover research hotspots of employee green behavior, many relevant keywords are taken to studied by the frequency and betweenness centrality.
6. To reveal the future research directions of employee green behavior, the analysis of burst terms is carried out.
7. To provide insights for future research by summarizing the enlightenment from the results of bibliometric analysis.

## 2 Brief background of the study

Green human resource management not only reduces the negative impact of corporate business activities on the environment, but also effectively improves core competitiveness. As policy executors in the enterprise green management process, employee behavior has a significant impact on green human resource management. A series of environmentally friendly behaviors by employees will help companies achieve sustainable development goals. Frontier research on employee green behavior has become a key part of promoting green human resource management.

From the perspective of management, economics, psychology and sociology, employee green behavior has multi-dimensional significance. From a management perspective, employee green behavior can be regarded as part of corporate social responsibility and a reflection of corporate culture and management practices. In modern enterprise management, guiding employees to form green work habits by formulating environmental protection policies, encouraging employees to adopt energy conservation and emission reduction measures, and conducting environmental protection training can not only enhance the corporate image, but also enhance the spirit of teamwork among employees. From an economic perspective, employee green behavior can help reduce a company’s operating costs. For example, behaviors such as saving water and electricity and reducing paper consumption may seem insignificant, but accumulated over the long term are not small savings for enterprises. In addition, as consumer awareness of environmental protection increases, the company’s green image can also attract more customers, thereby increasing market share. From a psychological perspective, the development of green behaviors is related to individual cognitive attitudes. Employees who realize the positive impact of their actions on the environment are more likely to continue to take green actions. Therefore, by commending green behavioral models and providing environmental protection knowledge, companies can effectively improve employee awareness of environmental protection and stimulate their intrinsic motivations. From a sociological perspective, employee green behavior reflects their sense of social responsibility. As society increasingly attaches importance to sustainable development, employee environmental behavior is not only a practice of personal values, but also a response to social expectations. Cultivating and strengthening this sense of social responsibility is of great significance for building a harmonious social environment. From the perspective of green human resource management practice, improving employee environmental awareness and promoting employee green behavior have become a key way for enterprises to efficiently implement green human resource management and promote sustainable development.

In order to promote sustainable development, Enterprises need the active cooperation of employees and their environmentally friendly behavior. A series of environmentally friendly behaviors by employees will help companies achieve sustainable development goals. Employee green behavior stems from the practice of environmental protection and aims to reduce negative environmental impacts through daily behaviors (Zhang et al., 2024; Mahmud, 2024). Employee green behavior not only includes environmental actions at the individual level, but also includes actions at the social level, such as supporting public transportation and reducing car use (Mahmud, 2024; Kaplan et al., 2024). Employee green behavior refers to a series of behaviors displayed by employees in an organization aimed at protecting the ecological environment and reducing the negative impact of personal activities on the natural environment (Zhang et al., 2024; Mahmud, 2024; Murillo-Ramos et al., 2024). These behaviors are not only an important supplement to the organization’s formal green management plan but also improve the efficiency of the organization’s green management measures and ultimately contribute to the sustainable development of the environment.

How to rely on employee behavior to achieve sustainable green operations has attracted increasing attention from academia and the industry. For now, the public’s awareness of green and low-carbon is gradually increasing, but the corresponding behavior lags behind. Inconsistency between awareness and behavior shows that there are still some obstacles to the realization of green behavior. As a key entity in achieving green and low-carbon goals, employee green behavior is not only a reflection of their green awareness, but also an active factor in achieving their goals. In recent years, research on the specific connotation of employee green behavior mainly includes the following representatives. Ahmed et al. (2024) believe that employee green behavior can help achieve the sustainable development goals of hotel organizations and point out a close relationship between employees’ environmental enthusiasm and specific green human resource management practices. Ma et al. (2024) explored how greenwashing affects employee green behavior and, in this context, studied the mediating role of green organizational identity and the moderating role of selfish leadership. The results showed that greenwashing negatively affects employee green behavior (Ma et al., 2024). Mohamad et al. (2024) management guidance for training programs can promote effective communication between direct supervisors and employees in sustainable organizations. Existing research results show that the continuous implementation of management guidance by direct supervisors who manage training activities in training programs can stimulate employee green motivation, thereby motivating employees to behave green (Mohamad et al., 2024). To comprehensively study the variables that affect employee green behavior from multiple perspectives, Feng et al. (2024) studied methods for determining key variables that affect employee environmental adoption behavior by explanatory structural models and MICMAC matrices. Research results indicate that employee motivation, values, and leadership are the most important variables affecting green behavior (Feng et al., 2024). Mohamed Azim and Abdul Mutalib (2024) used quantitative research methods to obtain the green behavior data of 545 employees in Malaysia’s manufacturing industry, and they used the data to explore the factors that influence green innovation. The research showed that green innovation is regulatory in employee green behavior. Tantawi and Noviana (2024) used quantitative methods to analyze the mediating role of perceived organizational support in the impact of employee green behavior on the sustainability of environmental performance and the moderating role of self-efficacy. Some scholars believe that organizational support does not mediate the impact of employee green behavior on environmental performance, while self-efficacy plays a role in strengthening pro-environmental behaviors in sustainable environmental performance (Tantawi and Noviana, 2024; Zhou and Zheng, 2024). Research on employee green behavior has important practical value. It will promote green transformation of enterprises, promote social and economic development, enhance employee green awareness, respond to environmental challenges and promote sustainable development.

## 3 Data source and search methodology

### 3.1 Data source

In this study, the data of bibliometric analysis is sourced from the Web of Science (WOS), which includes databases such as SCI-E, SSCI, A&HCI, and CPCI (Huang et al., 2022). WOS is a globally recognized academic research platform and encompass a wide range of highly reputable and influential academic journals (Huang et al., 2022; Shao et al., 2021). These databases of WOS provide detailed information on employee green behavior literature, including titles, authors, publication years, journal names, keywords, and abstracts (AlRyalat et al., 2019; İyibildiren et al., 2022; Shao et al., 2021). These all serve as critical foundations for subsequent analysis in this study.

WOS also offers a citation indexing feature that enhances researchers’ efficiency across various scientific literature retrieval, analysis, management, writing, and submission stages (AlRyalat et al., 2019; Huang et al., 2022; İyibildiren et al., 2022; Shao et al., 2021). The databases are updated weekly in WOS, ensuring the timeliness and accuracy of the information. The citation report provided by WOS reveals citation activities and trends, while analytical tools help identify research directions and patterns (Huang et al., 2022; Shao et al., 2021). WOS is employed to conduct the comprehensive bibliometric analysis in this study to identify the critical hotspots and emerging trends in the research field of employee green behavior.

### 3.2 Search methodology

CiteSpace has been used to analyze keyword frequencies, keyword clusters and mutation analysis by scholars (Demir et al., 2024; Sun et al., 2024; Wen et al., 2024). To analyze research frontiers and development trends, CiteSpace has become a powerful bibliometric analysis tool that helps researchers reveal hotspots, trends and frontiers in the research field (Demir et al., 2024; Sun et al., 2024). In order to analyze the trajectories, hotspots and trends of employee green behavior research, CiteSpace was used to carry out bibliometric analysis based on Web of Science in August 2024.

It is found that there is very little relevant literature on employee green behavior before 2010. Therefore, this study takes the relevant literature published from 2009 to 2024 as objects to conduct the bibliometric analysis. Research on employee green behavior originates from sustainable development. Therefore, the words related to sustainable development are used as keywords. The research content of literature types such as Article, Dissertation, and Proceedings Paper is usually relatively novel. Therefore, this study used these literature types as the search settings. Since citation rates vary by field and older papers are cited more than recent papers, the selection procedure for highly cited papers takes these factors into account. The first step is to count the number of papers cited at different levels of citation and construct distributions for each field and year. These distributions for each field or each year are then used to set selection thresholds by taking the same fraction of papers. According to the major purpose of this study, major rules and settings of search methodology are listed in Table 1.

**Table 1:** Major rules and settings of search methodology.

Index	Name	Content
1	Retrieval keywords	“employee green behavior,” “sustainability,” and “green development,” etc.
2	Retrieval Boolean operators	“AND,” “OR,” and “NOT”
3	Literature publication time	2009 to the present
4	Literature type	Article, Review, Dissertation, Meeting Abstract, Proceedings Paper, etc.
5	Literature language	English and Chinese
6	Wildcard rules	“behavior*” and “employee*”
7	Retrieval strategy	Topic “employee green behavior” AND Year Published “2009–2024,” Title “employee green behavior” AND Year Published “2009–2024,” Abstract “employee green behavior” AND Year Published “2009–2024,” Ttitle “employee green behavior,” etc.
8	WOS database	All Databases
9	WOS collections	All
10	Term source setting	Tile, Abstract, Author Keywords and Keywords Plus
11	Pruning setting	Pruning sliced networks
12	Node types setting	Term, Keywords, Reference, Author, Country, etc.
13	Term type setting	Burst Terms

A retrieval strategy using keywords such as “employee green behavior,” “green management,” “environmental responsibility,” “green economy,” “sustainability,” “environmental performance,” and “green development,” etc. is first established. Retrieval Boolean operators, including “AND,” “OR,” and “NOT,” are employed to narrow the search scope and ensure that the selected literature is closely related to the topic. To capture the dynamic changes in the research field of employee green behavior and reflect the contemporary research trends, the publication time of the literature search process is set from 2009 to the present. After data retrieval, a preliminary sorting and cleaning process is conducted to remove duplicate records and correct formatting errors, ensuring data quality. To further enhance the accuracy of the analysis, the literature is filtered by language. Finally, only the literature in English language is remained. It is made because English is the predominant language in global scientific research, and most high-quality academic work is published in English.

Keywords such as “employee green behavior,” “environmental performance,” and “pro-environmental behavior” are used for advanced searching. Because “behavior” is spelled in American English and “behavior” in British English, the specific search strategy in WOS is to search for titles containing “behavior*” and topics including “employee*. “The character “*” serves as a wildcard in the search process. In terms of topic search, “employee green behavior,” employee pro-environmental behavior,” “environmental organization citizenship behavior,” “environmental organization citizenship behavior,” and “employee environmental protection citizenship behavior,” etc. are taken to search the literature related to employee green behavior.

In this study relevant literature on employee green behavior research is used to build complex keyword network analysis to grasp research trends. The keyword search covers the perspectives of social responsibility leadership motivation and emotional factors. Based on the keyword analysis this study delves into the content information of published papers and summarizes existing research. A combination of keyword search and literature abstract analysis was used to reveal research trends in employee green behavior. After screening and removing irrelevant literature from the search results 788 relevant articles were obtained.

## 4 Trajectories in employee green behavior research

### 4.1 Brief review of publications

Research on employee green behavior focuses on various key areas, covering personal factors (e.g., environmental values, attitudes). Scholars have used a series of theoretical perspectives to study employee green behavior, such as planned behavior theory, social identity theory, and organizational support theory. Each of these frameworks provides unique insights into the drivers and processes behind environmental behavior in the organizational environment. From the perspective of the distribution of published journals, literature mainly published in the journals “Sustainability,” “Journal of Cleaner Production,” “Frontiers in Psychology,” etc. From the perspective of journal level, these journals are important journals in this research field. These journals are not only popular among researchers, but also have great appeal to readers. From the perspective of geographical distribution of literature, because the countries in Europe and North America have strong advantages in environmental awareness, policies, and social responsibility, scholars from Sweden, America, England and Germany have conducted in-depth research on employee green behavior. Globally, America is the first country to start studying employee green behavior. This is mainly because these countries have strong advantages in environmental awareness, policies, and corporate social responsibility. Enterprises in these countries have paid more attention to employee green behavior and formulated relevant policies and measures. In terms of the number of papers published by authors, Pacal Paillé published the largest number of relevant papers. His skills and expertise mainly include Human Resource Management, Organizational Theory, Environmental Management, etc.

According to existing research, individual characteristics fundamentally impact employee green behavior, while organizational green culture profoundly impacts it. Employee personal values and behavioral habits, as well as the environmental culture and policies advocated by their organizations, jointly affect employee green behavioral performance. Green human resource management practices such as promoting corporate environmental policies, conducting environmental training, providing formal and open communication platforms, and promoting and rewarding environmental behaviors, employee environmental awareness and skills can be enhanced, and they can be encouraged to show more green behaviors. The research results show that the feeling of excessive qualifications moderates the relationship between green human resource management practice and employee green behavior. This means that employee over-evaluation of capability affects commitment to environmental behavior.

In addition, previous studies have also pointed out that with employee education and knowledge improvement, the widespread sense of excessive qualifications in organizations and the importance enterprises attach to employee green behavior are important factors in promoting employees to show more green behaviors. By implementing measures such as green recruitment and providing environmental training, enterprises can effectively promote employee green behavior, thereby achieving the sustainable development goals of the enterprise.

To sum up, existing research on employee green behavior has revealed multiple influencing factors, providing the theoretical basis for corporate managers to encourage employees to demonstrate more environmental protection behaviors, promoting the green development of the organization and the sustainable development of society.

### 4.2 Distribution of publications by year

It is very important to understand the distribution of publications by year, because it reveals the evolution of subject knowledge over different time periods. As a quantitative analysis method, bibliometrics is often used to analyze indicators such as the number of academic publications, citations, and author cooperation networks to reveal the activity level, influence, and degree of knowledge innovation in the research field. The distribution of publications by year indicates research activity and interest in a particular area. Over time, an increasing trend in published literature typically signifies the growth of research activity and enhanced interest among scholars in that field. CiteSpace software systematically analyzes the published literature on employee green behavior. By examining the number of changes in published literature, this paper aims to reveal research trends and dynamics of the research on employee green behavior, providing insights for future research.

**Figure 1:**
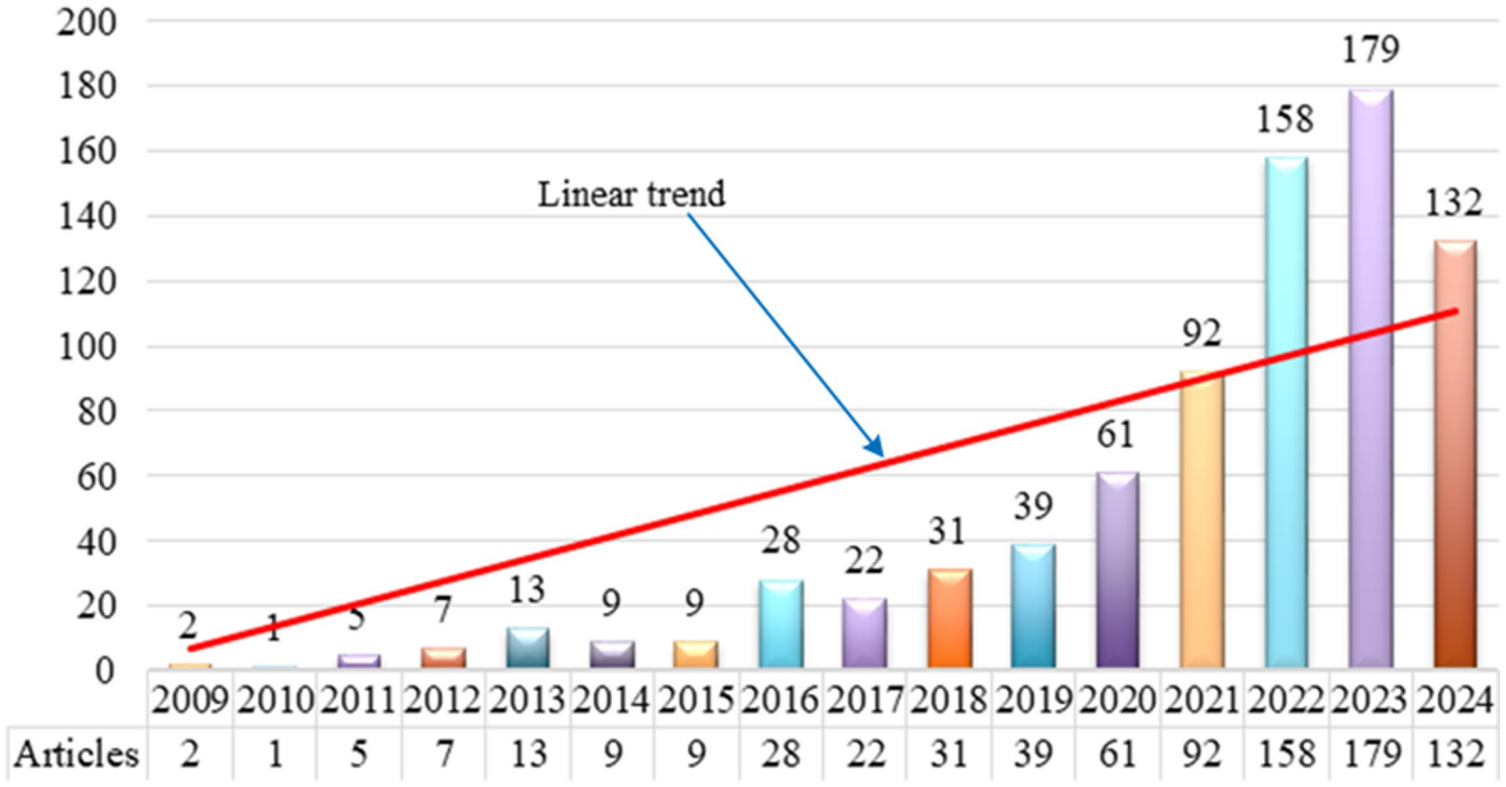
Distribution of publications by year from 2009 to 2024.

Research on employee green behavior has shown a significant upward trend and made many important contributions to enriching the theoretical development and practice of employee green behavior. Since 2018, research on employee green behavior has shown a rapid development trend. The distribution of publications by year from 2009 to 2024 is illustrated in Figure 1.

The publication trend of the research on employee green behavior from 2009 to 2024 is shown in Figure 1. It is seen that a total of 96 articles were published from 2009 to 2017, showing a fluctuating development trend. The relatively low number of publications during this period may be attributed to methodological limitations and the need for higher-quality relevant literature. After 2018, the number of published literature has gradually increased, showing consistent upward trends. After 2020, the number of published literature increased rapidly, reaching a peak of 179 articles in 2023. Since 2018, the research on employee green behavior has become more comprehensive and in-depth. As of August 2024, the number of published literature related to the research on employee green behavior has reached 132, which has exceeded half of the total published literature in 2023. The statistical results show that the publications on employee green behavior will continue to increase.

There is a close positive relationship between the number of published literature and the level of research activity. In the research field of employee green behavior, the annual increase in publications reflects scholars’ ongoing interest in the topic and indicates the level of research activity. This activity encompasses not only the deepening of theoretical research but also the exploration of new issues, the expansion of empirical studies, and the strengthening of interdisciplinary collaboration. These trends demonstrate researchers’ proactive response to global environmental challenges, aiming to enhance employee green behavior and drive corporate green transformation.

### 4.3 Distribution analysis of journals

Research hotspots not only attract widespread academic and public attention, but also usually attract the interest of many researchers, scholars and experts. They may focus on global challenges, research frontiers, emergencies, or major issues that have a broad impact on academia and society. Relevant research results are always published in major journals by researchers, scholars and experts. Analysis of the publications in major journals has become an important way to evaluate scholars’ attention.

After the dissertations and the conference papers are excluded, 721 journal articles are retained. For comparative analysis, journals with more than 25 publications were selected for statistical purposes. The journals “Sustainability,” “Journal of Cleaner Production,” “Frontiers in Psychology,” etc. are the leading journals on employee green behavior. Many influential papers on employee green behavior research are often published in these journals. The statistical results of the distribution of journals are presented in Table 2.

**Table 2:** Statistical results of the distribution of journals.

Journal name	Number	Nation	Percentage
Sustainability	106	Sweden	15.8
Journal of Cleaner Production	92	America	13.8
Frontiers in Psychology	83	Sweden	12.4
Corporate Social Responsibility and Environmental Management	79	England	11.8
International Journal of Environmental Research and Public Health	71	Sweden	10.6
Environmental Science and Pollution Research	62	Germany	9.3
International Journal of Hospitality Management	55	England	8.2
Journal of Sustainable Tourism	46	England	6.9
Business Strategy and the Environment	46	America	6.9
International Journal of Manpower	29	England	4.3

According to the data in Table 2, the number of publications in major journals shows a relatively concentrated trend. Among these leading journals, 669 articles were published, while the top five journals accounted for approximately 64.4% of the publication. This phenomenon reveals the triggers thinking about academic exchange models. The phenomenon that a large number of publications are published in a few journals is usually due to the high influence and authority of major journals. However, this phenomenon also has its negative effects. Because an excessively centralized publication model may hinder the generation of new ideas and opinions.

It is worth mentioning that the three journals “Sustainability,” “Journal of Cleaner Production,” and “Frontiers in Psychology” have published more than 80 articles, respectively, with specific numbers of 106, 92, and 83 articles. This data shows the influence of these journals in the academic community and reflects the current hot directions of academic research. “Sustainability” ranks first among all journals with 15.8% of articles, showing the importance and attention of the field of sustainable development. This concentrated trend in the number of publications is no accident. On the one hand, this may be related to the evaluation system of academic journals. In the current academic evaluation system, articles published in journals with high-impact factors often gain higher recognition. Therefore, researchers are more inclined to submit high-quality research results to these journals. On the other hand, leading journals are more likely to attract excellent research results due to their advantages in historical reputation, influence, and review quality, thus forming a positive cycle.

### 4.4 Distribution analysis of regions

The publication region distribution can reflect the importance and level of research on employee green behavior in different countries and regions. The publication regions are mainly concentrated in developed countries in Europe and North America. Among them, America is the first country to start studying employee green behavior. Scholars from America, England, Sweden and Germany have conducted in-depth research on employee green behavior, covering topics such as the cultivation of employee environmental awareness, the construction of corporate green culture, and the impact of employee green behavior on corporate performance. In addition, companies in these countries have also paid more attention to employee green behavior and formulated relevant measures to improve employee environmental awareness and behavior.

**Figure 2:**
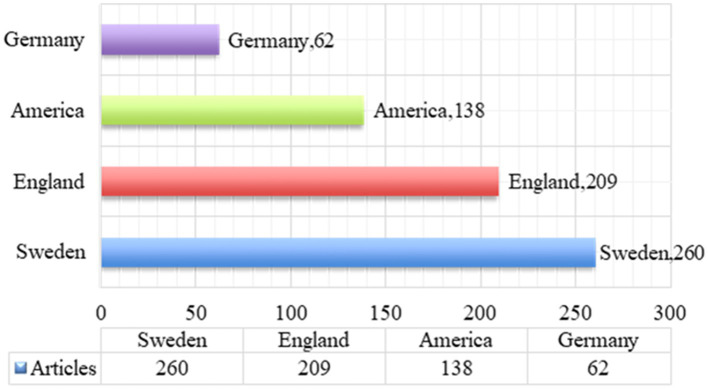
Statistics of regional distribution of publications.

By conducting statistical analysis of scientific paper publications in different regions, the intensity, field preferences, and academic influence of scientific research activities in each region will be better understood, thereby providing valuable information for policymakers, researchers, and academic institutions to promote the balanced development of scientific research and the optimal allocation of resources. The number of scientific papers published in each region can assess each region’s scientific research activity and research field preferences. Key regional research areas can be revealed by exploring the distribution of various disciplines in different regions. Through indicators such as citations and impact factors, the academic influence of scientific papers in various regions and their status in international and domestic academic circles can be evaluated. Through the above analysis, we can have a clearer understanding of the current situation and potential of different regions around the world in employee green behavior research and provide the necessary scientific basis for further research on employee green behavior and future practice of green human resource management. Analyzing publication quantities and the countries of journals provides insights into the distribution of publications by region, with the results summarized in Figure 2.

Figure 2 shows that Sweden has the highest number of publications, with 260 papers, followed by England with 209 papers, America with 138 papers, and Germany with the fewest at 62 papers. According to the statistics presented in Figure 2, Sweden accounts for the largest publications share at 39%, whereas Germany has the smallest share at 9%. These statistics indicate that Sweden highly emphasizes research into employee green behavior. The data presented in Figure 2 further reflects the level of activity and contribution of different countries in academic publishing. Sweden leads in the number of publications, indicating a high level of activity and output in educational research and publishing within the country. England follows closely, suggesting a solid presence and significant contribution to academic publishing. As a central hub for global research and innovation, America ranks third in publication volume, demonstrating its considerable strength and influence. Conversely, Germany has the lowest number of publications, which may reflect a relatively lower activity level in academic publishing. The data presented in Figure 2 provides important reference points for understanding the contributions of different nations in scholarly publishing. According to America, England, Sweden and Germany are the world’s major developed countries and the data shown in Figure 2, we can believe that economically developed countries are more concerned about employee green behavior.

### 4.5 Influence analysis of main authors

The influence of the main authors is not only reflected in academic citations, but also more deeply affects the formulation of green strategies of enterprises. The research results of the main authors not only play a leading role in the academic development track, but also profoundly affect corporate practice. High-impact authors are often better able to promote the implementation of green concepts in enterprises, which may be related to the ideas they emphasize on sustainable development. From early theoretical research to recent empirical analysis, these authors have not only deepened our understanding of the topic, but also expanded new research directions.

According to the results of the CiteSpace collaboration network knowledge map analysis, the collaboration network among the leading authors in employee green behavior research is relatively dense, with a significant cluster formed by crucial scholars. The largest collaborative network comprises scholars such as Guiyao Tang, Pascal Paillé, Hannes Zacher, Heesup Han, and Bilal Afasar. This paper ranks the number of publications by the authors and lists statistics for the top five authors, as shown in Table 3.

**Table 3:** Statistics for top five authors on the number of literature published.

Author	From year	To year	Number
Pacal Paillé	2012	2024	14
Heesup Han	2019	2024	12
Guiyao Tang	2015	2024	10
Hannes Zacher	2012	2024	9
Bilal Afasar	2016	2024	9

Based on the statistical results presented in Table 3, Pascal Paillé ranks first among the authors with a substantial publication volume, having published 14 papers. Meanwhile, Hannes Zacher and Bilal Afasar ranked fourth and fifth, each having published 9 papers. From 2012 to 2024, among the top five authors in the number of papers published, each author published an average of about 11 papers. In addition, in the 12 years from 2012 to 2024, 54 papers were published by the top five authors. The top five authors publish an average of about 5 articles per year between 2012 and 2024. Paillé and Mejía-Morelos (2014) pointed out that employees were more willing to perform green discretionary behaviors when they sought benefits of having greater green absorptive capacity. Paillé et al. (2022a, 2022b) reported that perceived organizational support for the environment was a mediator between green rewards and employee environmental performance. Paillé et al. (2022a, 2022b) believed that employee engagement was vital when achieving corporate environmental sustainability. Manosuthi et al. (2024) believed that different combinations of moral norms, organizational support, psychological ownership, past green behavior, and green practice behavior could shape employee behavior to perform green practices at work. Tang et al. (2017) believed that employee performance is evaluated for set performance indicators in activities related to fulfilling the ecological objectives. Methods for measuring variables of green human resource management proposed by Tang are widely adopted by researchers in today.

**Figure 3:**
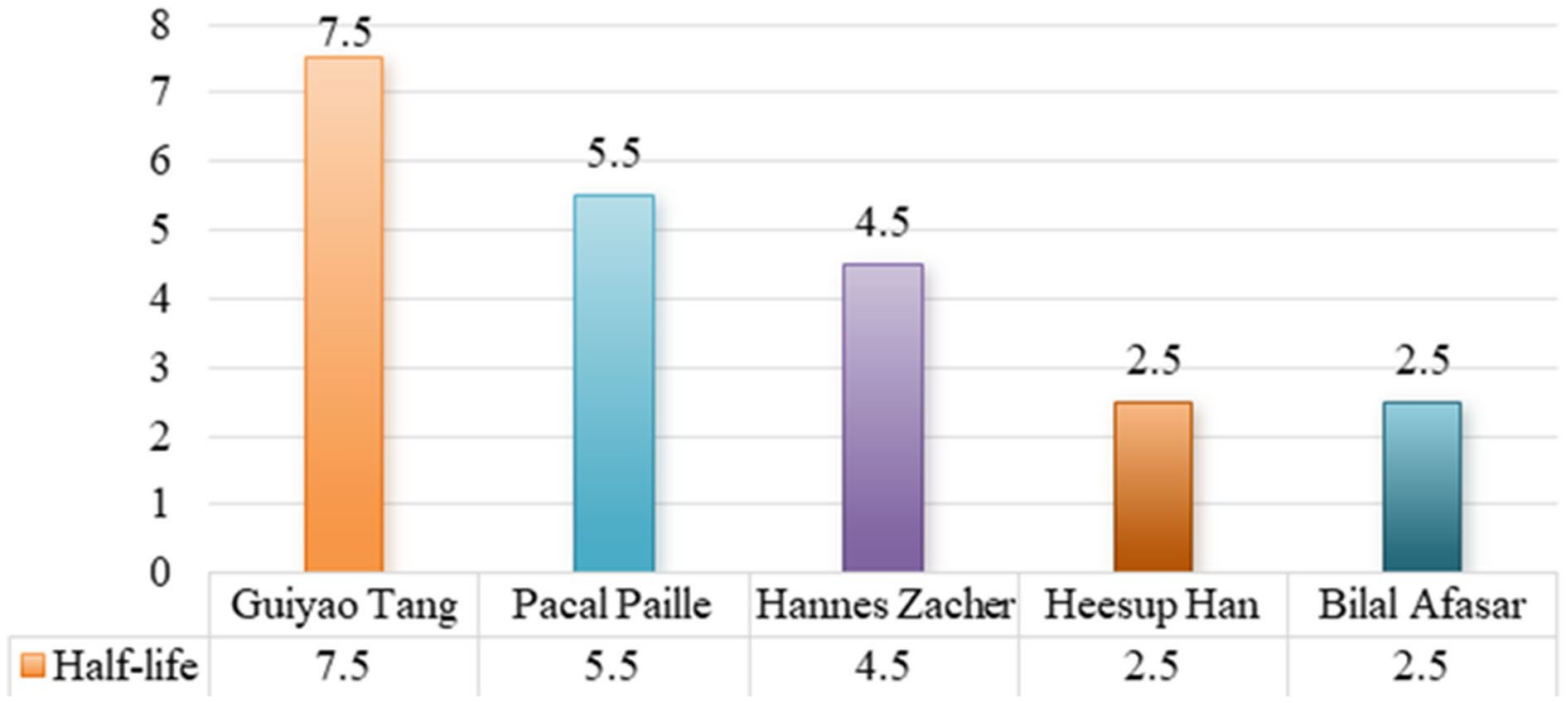
Literature half-life of top 5 authors.

The literature half-life is an important indicator reflecting the speed of knowledge update. It is often used to assess the life cycle of the novelty and importance of research results. If research results are continuously cited, it means that such research results have certain influence. On the contrary, if the research results are quickly forgotten, it means that the research direction has been improperly chosen or the quality of the research needs to be improved. According to the results from CiteSpace, literature half-life of top 5 authors is shown in Figure 3.

The half-life represents the aging of the literature, with a higher value indicating a more significant impact. Based on the results shown in Figure 3, the half-life values are 7.5, 5.5, 4.5, 2.5, and 2.5, corresponding to Guiyao Tang, Pascal Paillé, Hannes Zacher, Heesup Han and Bilal Afasar, respectively. The half-life values for these leading scholars indicate that the literature in employee green behavior research generally has a longer impact duration. This implies that research has a significant impact in this field and that the literature has a lasting influence. For example, although methods for measuring variables related to green human resource management and employee green behavior have been proposed by Guiyao Tang for several years, they are still widely adopted by scholars. The top five authors in the number of papers published have an important impact on the research of employee green behavior.

## 5 Research hotspots and trends

### 5.1 Research hotspots

The topics of investigation by experts and scholars are known as research hotspots, and these can help predict future research directions. In academic research, keywords are the eyes of the paper and can reflect the research theme and core content of the article. As the keywords in the core part of the article, they can best represent the research direction and research content of the entire paper. Keywords are like the ID card of an article, allowing readers to know at a glance what the article is about. In the world of scientific research, every keyword is the beginning of a story, and every research hotspot is the starting point of a journey. Keywords are the core terms that encapsulate the themes, methods and conclusions of literature. They accumulated over time in a specific research field can reveal the overall characteristics, content connections, developmental trajectories, and research trends. To analyze the research hotspots of employee green behavior, a bibliometric analysis is carried out by the CiteSpace with relevant keywords.

**Figure 4:**
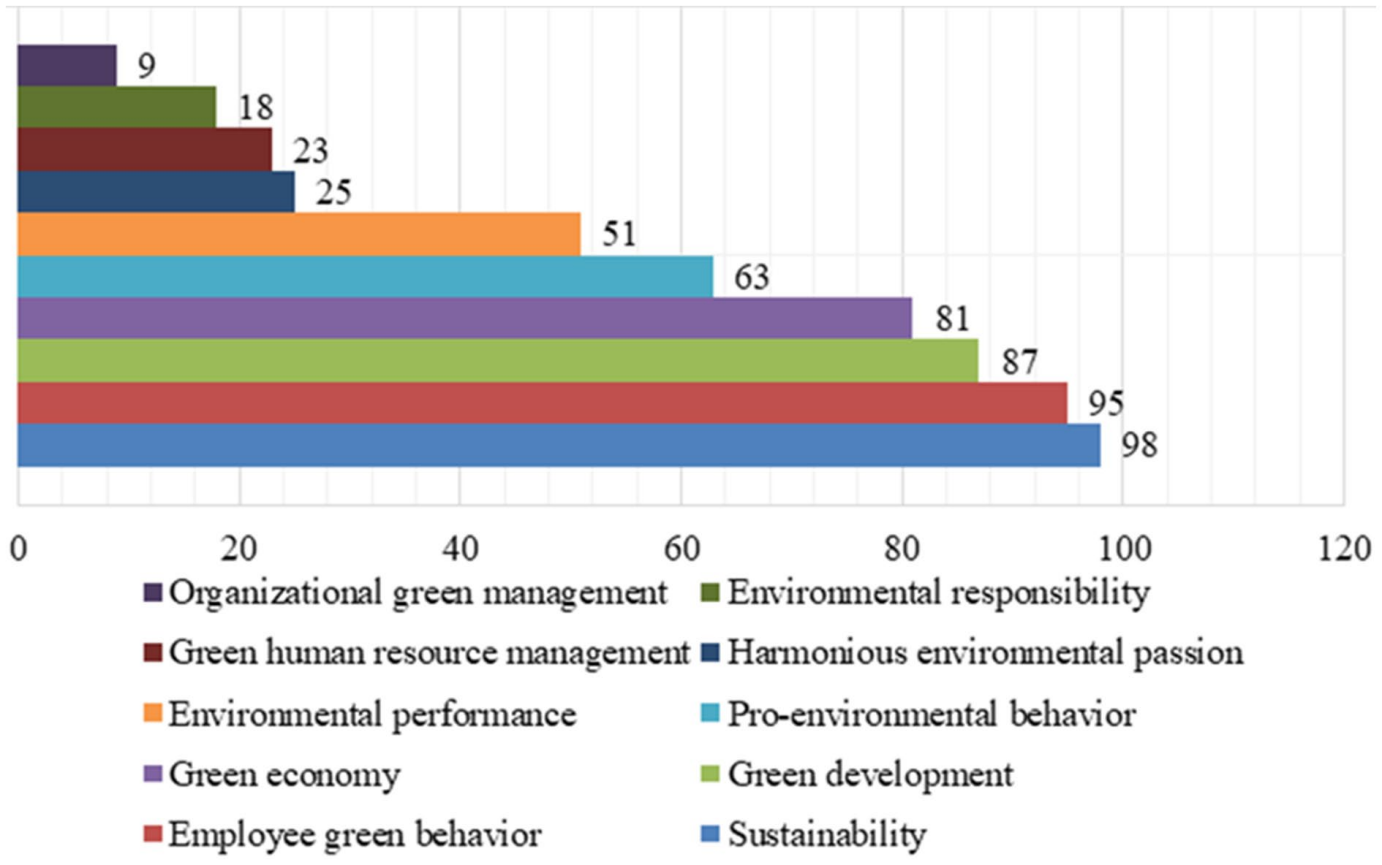
Statistics of frequency for top 10 keywords.

When using keyword analysis to research hot topics, the diversity of keywords is a principle that cannot be ignored. Since employee green behavior is a multidisciplinary field covering many disciplines such as psychology, management, and environmental science, when selecting keywords, we should try to include the perspectives and terms of these different disciplines. To discover the major research directions of employee green behavior, keywords such as “sustainability,” “employee green behavior,” “green economy,” “green development,” and “green human resource management” etc. are used to analyze the hotspots of research on employee green behavior by this paper. Based on the analysis results from CiteSpace, the statistics of frequency for top 10 keywords are shown in Figure 4.

Figure 4 shows that the frequency of five keywords “sustainability,” “employee green behavior,” “green development,” “green economy,” and “pro-environmental behavior” ranks among the top five, reaching 98, 95, 87, 81, and 63, respectively. The keywords and their frequency reveal the current society’s attention to environmental issues and reflect the main research hotspots of employee green behavior. These high-frequency keywords reflect a core concept: environmental protection and economic and social development are not opposed but can promote each other. By promoting sustainable development, encouraging employee behaviors, implementing green development strategies, building a green economic system, and popularizing pro-environmental behaviors, we can create a more prosperous, healthier, and harmonious world together.

Environmental issues have increasingly become a hot topic of public attention. The concept of sustainable development emphasizes meeting the needs of contemporary people without compromising the ability of future generations to meet their needs. This concept emphasizes the effective use of resources and long-term protection of the environment. High-frequency keywords such as environmental protection, economic development and social development reflect the mutually reinforcing relationship between these areas. By promoting sustainable development, encouraging employee behavior, implementing green development strategies, building a green economic system, and popularizing environmental behavior, we can jointly create a more prosperous, healthier and harmonious world.

Sustainable development is a development concept that aims to meet the needs of current generations while protecting the interests of future generations. This concept has been widely recognized and supported. Employee environmental awareness is crucial to the sustainable development of the company. Encouraging employees to adopt environmentally friendly behaviors, such as reducing waste and saving energy, can improve a company’s environmental performance. Some companies have begun to take measures to promote employee environmental behavior, such as providing training courses and establishing reward mechanisms. These measures can not only increase employees environmental awareness, but also enhance their corporate identity.

**Figure 5:**
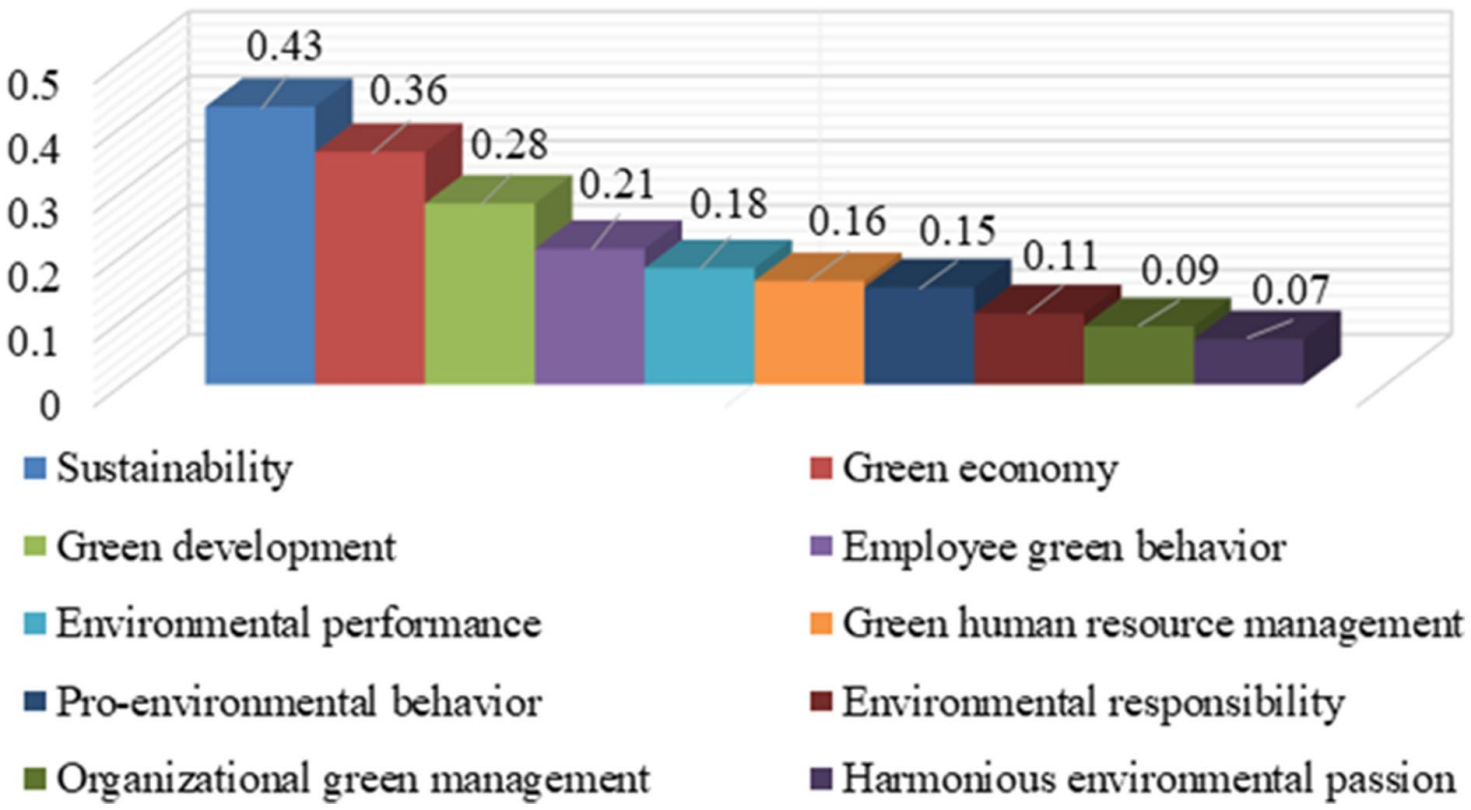
Statistics of betweenness centrality for top 10 keywords.

There is a close connection between betweenness centrality and research hotspots. Researchers or institutions with high betweenness centrality are more likely to have access to new research trends, and thus participate in the formation and development of research hotspots earlier. Through cooperation and exchanges with other researchers, they can promote the spread of hot topics to a wider field. On the other hand, research hotspots will attract the attention of many researchers, form a dense communication network, further promoting the intermediation centrality of some of these nodes. By applying betweenness centrality, researchers can better grasp scientific research trends and provide valuable guidance for future research directions. A higher value of betweenness centrality indicates a more significant mediating role for the keyword. Betweenness centrality has been an key way to discover the importance of keywords. Based on the results from CiteSpace, the statistics of betweenness centrality for top 10 keywords are shown in Figure 5.

According to the analysis results shown in Figure 5, the betweenness centrality of keywords “sustainability,” “green economy,” “green development,” “employee green behavior,” and “environmental performance” ranks among the top five, reaching 0.43, 0.36, 0.28, 0.21 and 0.18, respectively. The analysis results show that these five keywords play an important role in the research field of employee green behavior. The betweenness centrality value of the keyword “sustainability” tops the list, reaching 0.43. It not only reflects its core position in the field of green research but also shows that the concept of sustainable development has become an indispensable part of enterprise strategy. The betweenness centrality of the keywords “green economy” and “green development” is 0.36 and 0.28, respectively. Although the two words are slightly different in the face, they both point to the same goal of promoting economic growth while protecting the environment. A green economy emphasizes the effective use of resources and environmental sustainability in economic activities. Green development is a more comprehensive development model focusing on economic growth and harmonious development in many aspects, such as society and culture. In this context, employee green behavior is particularly critical because it is an important driving force for the enterprise to achieve the goal of green development.

The keyword “employee green behavior” ranks fourth in betweenness centrality. It reveals that employee environmental awareness and green behavior play an important role in achieving sustainable development of enterprises. From small daily measures to save energy and reduce emissions to participating in the company’s environmental protection projects, every employee’s green behavior contributes to the company’s sustainable development. The keyword “environmental performance” ranks fifth in terms of betweenness centrality. Good environmental performance not only enhances the social influence of enterprises but also brings more economic benefits to enterprises. Therefore, how to optimize environmental performance by improving employee green behavior has become a common concern for researchers and business managers.

From an environmental performance perspective, the relationship between employee green behavior and environmental performance is not always as direct as expected. For example, if employees actively participate in waste sorting and recycling, they can reduce the pressure on the company’s waste disposal, thereby reducing environmental costs. However, this simple linear relationship does not always hold true. Because environmental performance is a complex systematic indicator that is affected by many factors, including policies, regulations, markets and technologies. For example, when a company promoted garbage classification, although the employees were very cooperative, they found that the classified garbage still needed to be treated uniformly and did not substantially reduce the treatment cost. In this case, although employee green behavior enhances the company’s image of environmental protection, its direct impact on environmental performance is limited.

### 5.2 Research trends

**Figure 6:**
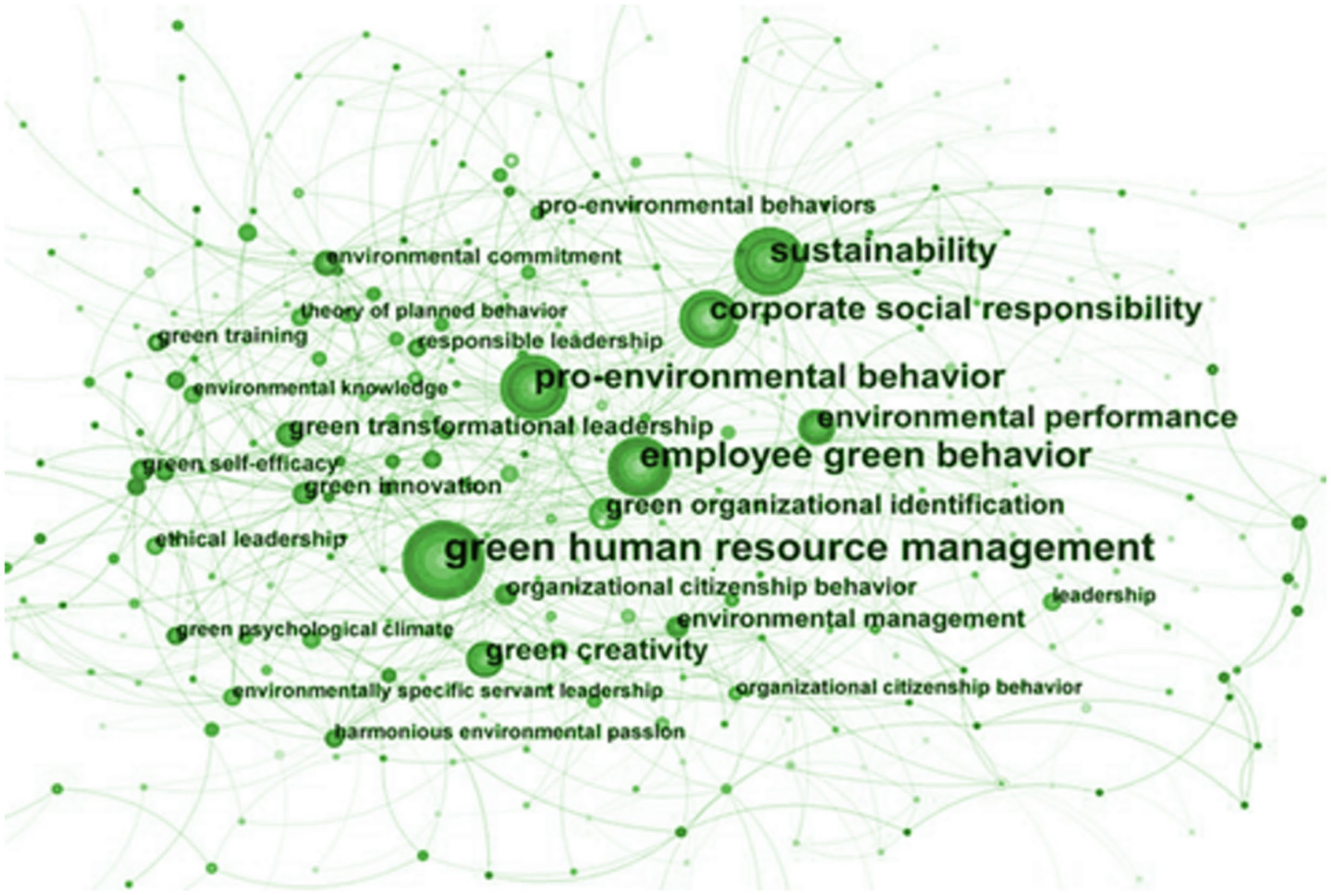
Keywords co-occurrence map.

Keyword co-occurrence analysis can illustrate the hotspot changes and the analytical perspectives over different periods by representing the keywords as nodes. The size of the nodes and text will indicate the frequency of keyword occurrences. The lines connecting nodes represent relationships established over different periods, with line thickness and density reflecting the strength of keyword co-occurrence. Based on the analysis results from CiteSpace, the keyword co-occurrence map is shown in Figure 6. According to the keyword co-occurrence map from the literature, 400 high-frequency keywords are identified, forming 854 connections. Among them, node “green human resource management” has the largest number of connections, while nodes “employee green behavior” and “sustainability” have the second largest number of connections.

The keywords such as “green human resource management,” “sustainability,” and “corporate social responsibility” appeared earlier. In contrast recent years have seen the emergence of keywords related to interdisciplinary research in psychology and management such as “harmonious environmental passion” and “green psychological climate,” which may represent the latest research directions in employee green behavior. The keywords like “employee green behavior,” “leadership,” and “green human resource management” appeared earlier in the statistical period. Starting in 2019 new keywords such as “ethical leadership,” “trust,” and “green innovation” have begun to emerge indicating that researchers are increasingly focusing on how to improve leadership and optimize organizational environments to promote employee green behavior and guide green innovation.

The analysis results from CiteSpace revealed several terms worthy of attention: green self-efficacy, employee well-being, and green psychological climate. These terms may represent a new research trend in the future. An employee with a high sense of green self-efficacy will actively seek and practice ways to save energy and reduce emissions at work, thereby promoting the overall green development of the company. The cultivation of self-efficacy requires efforts of enterprises through various aspects such as training, incentive mechanisms and cultural construction. Employee well-being focuses on employee job satisfaction and their perception of the working environment. A healthy and green office environment can significantly improve employee happiness, thereby increasing work engagement and efficiency. Enterprises can create an environmentally friendly and comfortable working environment by introducing green plants and reducing pollution sources. Green psychological climate describes collective attitude on environmental protection within the enterprise. The formation of green psychological climate requires the exemplary role of leaders and the participation of all employees. The appearance of burst terms such as “green self-efficacy,” “employee well-being,” and “green psychological climate” also indicate that research on employee green behavior will pay more attention to employee consciousness and psychological rewards in the future.

## 6 Discussion

In order to analyze trajectories, discover hotspots, reveal trends and provide insights for future research in employee green behavior research, a bibliometric analysis is carried out in this study. Based on the analysis result, it is necessary to discuss some important findings in this study.

1. From the perspective of distribution of publications by year, publications have gradually increased since from 2018. This trend indicates that topics related to employee green behavior are of increasing interest to researchers. It also indicates that scholars are trying to find new efficient ways to promote employee green behavior. From the deeper perspective of social development, which in recent years is facing major challenges related to this research area, there is an urgent need for solutions related to employee green behavior.
2. From the perspective of regional distribution, developed countries attach more importance to employee green behavior research. Most of the literature comes from four countries: America, England, Sweden and Germany. Among them, Sweden has contributed the most to employee green behavior research. Why developed countries are more concerned about the green behavior of their employees. Are developing countries facing similar issues. It is worthwhile to further study the social development trajectory behind this phenomenon.
3. From the perspective of the distribution journals, a large number of publications are published in a few journals. It suggests that triggers thinking about academic exchange models. In addition to the high influence and authority of major journals, this phenomenon deserves our attention to the research frontiers in the current field and the issues highlighted in the social development process. Publishing research results in top journals is everyone’s dream. However, the possible waste of academic resources behind this phenomenon deserves our consideration. We should consider whether it is reasonable for a large number of research resources to focus on certain topics.
4. The appearance of burst terms such as “green self-efficacy,” “employee well-being,” and “green psychological climate” represent new trends of employee green behavior research. The sudden proliferation of these terms suggests that relevant research is becoming the focus of research on employee green behavior. Relevant researchers and business managers should pay more attention to research trends and research results in these aspects. The emergence of these terms also means that employee green behavior research has risen to a new stage and has encountered new problems in the new stage. Combined with the current context of social development, employee happiness is a rewarding concern for most people.
5. The emergence of concepts such as green self-efficacy, employee well-being and green psychological climate not only focus on the relationship between individuals and the environment, but also involve the psychological climate within the organization and the well-being of employees. These concepts are not only related to employee individual development and team collaboration, but are also closely linked to the sustainable development strategy.
6. Future research on employee green behavior may focus on green self-efficacy, employee well-being and green psychological climate, which require companies to make systematic adjustments such as strategic planning, organizational structure, and cultural construction. However, differences in values and lifestyles of different employees also make it difficult to build a unified psychological climate. Issues such as how to balance work and life, how to deal with work pressure, and how to establish a fair promotion system are all important factors affecting employee well-being.

## 7 Conclusion

Research on employee green behavior is a multidisciplinary field encompassing various areas such as business management, environmental science, psychology, and sociology. Research hotspots, research trends and research trajectory are the key foundation for further research on issues related to employee green behavior. To obtain the research trajectory and research trends of employee green behavior, CiteSpace software is used to perform analysis of literature published from 2009 to 2024. Based on the results of bibliometric analysis in this study, it leads to the following findings.

1. Distribution of publications related to employee green behavior by year from 2009 to 2024 has shown increasing trends. The annual increase in publications on employee green behavior reflects that scholar are ongoing interest in the topic. The increasing trends of publications indicate that the researchers are trying to find efficient measures to enhance employee green behavior. The increasing of publications is undoubtedly a remarkable phenomenon. It not only reflects the vigorous development of employee green behavior research, but also reflects the high level of activity in the field of employee green behavior research.
2. The journals “Sustainability,” “Journal of Cleaner Production,” “Frontiers in Psychology,” etc. accounted for 64.4% of the publication, which shows that the number of publications in major journals shows a relatively concentrated trend. It reveals the triggers thinking about academic exchange models. The phenomenon of literature publication concentrated in a few journals is the result of the combined action of multiple factors. These major journals usually have high influence and authority and can attract more readers and citators. However, this concentration may also lead to homogenization of academic research, as researchers may excessively pursue publishing in major journals while neglecting research in other fields. This concentration may also have a negative impact on the development of academia, as it may hinder the generation of new ideas and perspectives.
3. Publishing regions of literature on employee green behavior are mainly concentrated in Europe and North America. Sweden has the highest number of publications, followed by England, America, and Germany. Sweden accounts for the largest publications share at 39%, whereas Germany has the smallest share at 9%. In terms of geographical distribution of literature on employee green behavior, because the countries in Europe and North America have strong advantages in environmental awareness, policies, and social responsibility, scholars from Sweden, America, England and Germany have conducted in-depth research on employee green behavior. Enterprises in these countries have paid more attention to employee green behavior and formulated relevant policies and measures.
4. The largest collaborative network comprises scholars such as Guiyao Tang, Pascal Paillé, Hannes Zacher, Heesup Han, and Bilal Afasar. The analysis results of the top five published papers show that these scholars have frequent research activities in employee green behavior research and have made great contributions to the field. The main authors of the published literature have proposed theories and methods with research value and practical guiding significance. Although the methods proposed by some leading authors have been around for many years, they are still adopted and cited by many scholars today.
5. The frequency of the five keywords “sustainability,” “employee green behavior,” “green development,” “green economy,” and “pro-environmental behavior” ranks among the top five, reaching 98, 95, 87, 81, and 63, respectively. The frequency of the five keywords reveal the society’s attention to environmental issues and reflect the main research hotspots of employee green behavior, reflecting the factor that environmental protection, economic development and social development can promote each other.
6. The betweenness centrality of keywords “sustainability,” “green economy,” “green development,” “employee green behavior,” and “environmental performance” ranks among the top five, reaching 0.43, 0.36, 0.28, 0.21, and 0.18, respectively. The betweenness centrality of keywords “sustainability,” “green economy,” “green development,” “employee green behavior,” and “environmental performance” indicate that employee green behavior is particularly critical because it is an important driving force for the enterprise to achieve the goal of green development. Improving employee green behavior has become a common concern for researchers and business managers.

From the perspective of the issues focused on in the existing literature, the existing literature mainly taken situational factors as the core and studied on employee green behavior from green environmental climate, psychological climate, corporate green human resource management, etc. The existing literature mainly taken the positive benefits of employee green behavior on enterprise development and the environment protection as the background, and studied the positive effects and rewards of employee green behavior from the aspects such as psychological factors, environmental protection effects, management performance, and mental happiness. However, the existing research lacked discussions on how the employee green behavior will appear in the specific results of enterprise development, and how the employee green behavior specifically will affect the sustainable development of enterprises. In the face of increasingly serious global environmental problems, companies are beginning to realize the importance of sustainable development. Employee green behavior is undoubtedly an important force in promoting enterprises to become green and achieve sustainable development. However, when we take an in-depth look at the existing research results, it is not difficult to find a significant gap-there seems to be a lack of in-depth discussions on how employee green behaviors translate into visible results.

As the discussion of the green concept continues to be deepen, this study believes that the research on employee green behavior will be further developed and improved in several key areas. First, exploring employee green behavior theories in specific local workplaces is recommended to enrich the theoretical framework of employee green behavior. Secondly, integrating the impact of information technology into the organizational environment will provide an in-depth understanding of employee green behavioral intentions and influencing factors. Third, the researchers should further research defining employee green behavior by examining the factors and effectiveness that affect the specific type of green behavior. Finally, multi-perspective analysis should be adopted to promote employee green behavior research.

As a literature analysis tool, CiteSpace is widely used in the exploration of knowledge and cooperative relationships in the scientific field due to its powerful functions. However, despite its powerful capabilities, we cannot ignore some limitations of CiteSpace, such as incomplete data source coverage, insufficient update speed, and difficulty in handling interdisciplinary research. These issues may affect the accuracy and depth of research results. CiteSpace data sources mainly come from the Web of Science, a database containing a large number of academic journal articles. However, Web of Science does not cover all journals in all fields, especially for certain social sciences and humanities journals, where coverage may be low. Therefore, when researchers use CiteSpace for thematic or collaborative network analysis, they may miss some important information due to the incomplete data sources. In order to more accurately obtain the research trends and development trajectory of employee green behavior, more perspective analysis and research are necessary. In the future, this study will further research much more methods to obtain research trends and research trajectories of employee green behavior.
